# Work-Up of Lower Extremity Swelling in a Patient With Ehlers-Danlos Syndrome

**DOI:** 10.7759/cureus.27973

**Published:** 2022-08-13

**Authors:** Karim A Zaazoue, Jacob M Core, Ahmed Altan, Mohamed A Elboraey, Charles A Ritchie

**Affiliations:** 1 Interventional Radiology, Mayo Clinic, Jacksonville, USA; 2 Interventional Radiology, Moffitt Cancer Center, Tampa, USA

**Keywords:** vascular complications, endovascular embolization, soft tissue tumors, pseudoaneurysm, ehlers-danlos syndrome

## Abstract

A 36-year-old woman with Ehlers-Danlos syndrome (EDS) presents with a painful and enlarging right lower extremity mass prompting imaging work up. Herein we present a case report of an uncommon complication and a unique treatment option of a large right anterior tibial artery pseudoaneurysm caused by repetitive microtrauma in a patient with EDS and a congenital club foot.

## Introduction

Ehlers-Danlos syndrome (EDS) is a connective tissue disorder characterized as impaired collagen metabolism, predominantly involving joints, skin, and blood vessel wall [1]. EDS can be classified into 13 different types based on 20 unique gene mutations, and varying clinical presentations. The most common clinical presentation includes hypermobility of the joints, hyperelasticity of the skin, and a varying degree of tissue fragility [1]. Vascular complications can arise from EDS, more commonly with vascular EDS (Type IV), and can include rupture of major blood vessels and organs [2].

The differential diagnosis for an enlarging lower extremity mass or swelling is broad, but includes muscle spasm or herniation, deep venous thrombosis, edema or hematoma. However, soft tissue tumors such as liposarcoma, angiosarcoma, and undifferentiated pleomorphic sarcomas should also be considered. This case report summarizes the clinical and imaging work-up of a young woman presenting with a painful, palpable mass of the right lower extremity.

## Case presentation

A 36-year-old female patient presented with an enlarging right lower extremity that was tender and had a reported palpable mass on the anterolateral aspect of the right calf. Pertinent past medical history includes EDS (type unknown), spontaneous colon perforations, and right clubfoot deformity corrected with a rigid lower leg brace. She denies a history of trauma to this region, and has not experienced fever, chills, weight loss or previous episodes of spontaneous hemorrhage or bleeding.

On physical examination, the palpable abnormality is located adjacent to the contact site of the splint she wears for the club foot deformity that provides static dorsiflexion assistance and lateral stability for the entire foot-ankle area.

**Figure 1:**
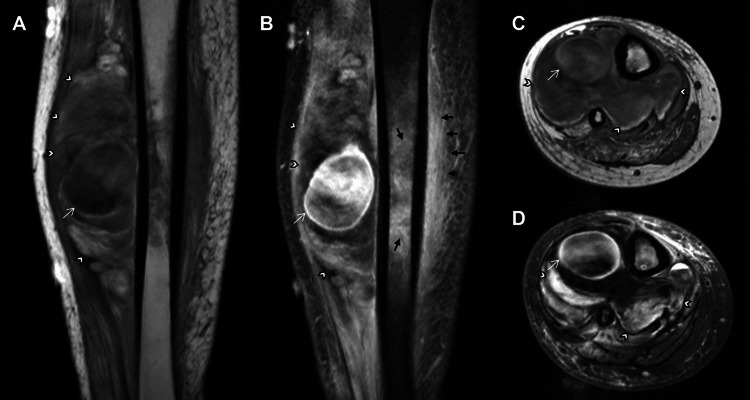
Right Lower Extremity MRI showing pseudoaneurysm (A) Coronal pre-contrast, (B) Coronal post-contrast, (C) Axial pre-contrast, and (D) Axial post-contrast T1W MRI showing 3.9 cm pseudoaneurysms (white arrows) in the anterolateral compartment of the right lower extremity calf. There is a sizeable hematoma (white arrowheads) with surrounding soft-tissue enhancement and intramedullary enhancement (black arrows) in the subjacent tibial diaphysis. Note: Pulsation artifact is observed within the pseudoaneurysm.

Contrast-enhanced magnetic resonance imaging (MRI) of the right lower extremity demonstrated a 3.9-cm pseudoaneurysm (PSA) in the anterior compartment with pulsation artifact and surrounding hematoma measuring up to 13.9 cm (Figure 1). Also noted was a 7-cm segment of intramedullary enhancement affecting the adjacent tibia with surrounding nodular and enhancing soft tissue.

An ultrasound-guided biopsy was performed of the nodular soft tissue to exclude an underlying neoplasm. Pathology demonstrated an acute inflammatory process and small fragment of reactive bone without evidence of malignancy.

**Figure 2:**
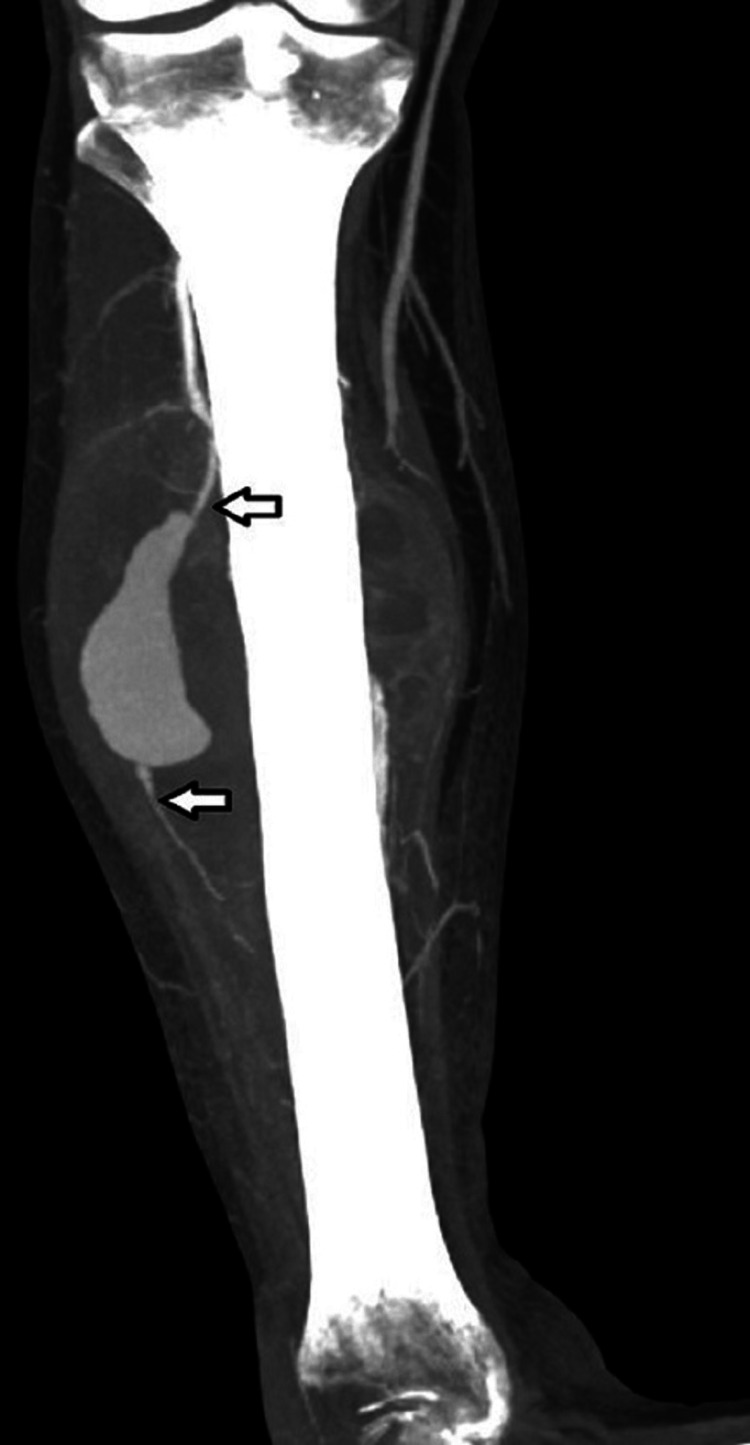
Pseudoaneurysm CT angiography of the right lower extremity confirming the pseudoaneurysm arising from the proximal aspect of the anterior tibial artery (arrows).

Given the concern for a PSA in this patient, a computed tomography angiogram of the abdomen and pelvis with lower extremity run off (Figure 2) was performed which revealed the PSA arising from the proximal segment of the anterior tibial artery (AT). Several other fusiform true aneurysms were identified in visceral arteries, the right common femoral artery, and the right peroneal artery.

The patient was referred to Interventional Radiology for embolization of the PSA once malignancy was excluded. Surgical management was deferred as the patient was only given the option for amputation and was told that there was a high risk of poor wound healing following surgery. As an alternative to amputation, endovascular embolization was offered.

**Figure 3:**
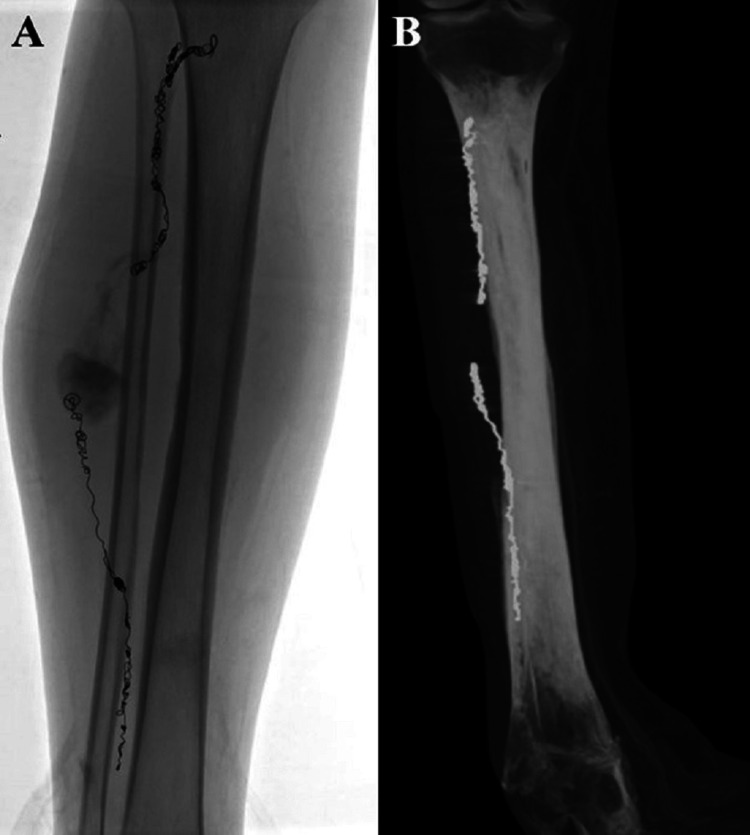
Post embolization and at eight months follow-up A. Spot fluoroscopic image showing endovascular embolization in proximal and distal anterior tibial artery, as well as stagnant contrast in the aneurysm sac. B. CT angiography performed eight months after intervention showing no contrast filling of the pseudoaneurysm.

The endovascular embolization was performed through direct access into the anterior tibial PSA and a bidirectional embolization of the anterior tibial artery. Proximal and distal anterior tibial artery embolization occurred through retrograde and antegrade manipulation. This approach was opted to avoid potential complications from manipulations of non-target vessels in this patient with multiple additional aneurysms in the right lower extremity attributed to the patient’s EDS. No coils were placed in the pseudoaneurysm to avoid a large coil mass which causes late complications. Stagnant contrast was noted in PSA at the end of the procedure (Figure 3A). Surveillance computed tomography angiography at eight months demonstrated no residual filling of the pseudoaneurysm (Figure 3B).

## Discussion

The clinical evaluation and workup of a patient with EDS who presents with a painful lower extremity mass in the absence of trauma or clinical evidence of blood loss can be difficult. However, a degree of suspicion is warranted as spontaneous lower extremity pseudoaneurysm has been reported in the literature [3]. Vascular events stemming from underlying EDS are well-known across several types, though typically much more serious and more common when associated with type IV. Spontaneous rupture can be the first presenting sign, though PSA formation can remain subclinical for unknown periods of time [4].

Of the 13 variants, only four types can be confirmed by laboratory tests. This makes the classification of EDS a challenge in most cases [5]. In a previous literature review of 31 patients with vascular EDS, Cikrit et al. found that only five patients presented with symptoms suggestive of EDS prior to the vascular event [2].

When present, a pulsation artifact centered at the pseudoaneurysm can be a helpful clue on MRI. This is especially important as the degree of surrounding soft tissue enhancement and abnormal signal may lead to a broadened differential diagnosis, including a soft tissue mass, as demonstrated in the present case [6].

Due to vessel fragility, both surgical and endovascular procedures should be handled with care in this patient population. The rate of complications for endovascular procedures was found to be as low as 2% in a single-institution retrospective study [7]. Comparatively, complications for surgical procedures were found to be 33% in another retrospective study by Brooke et al. [8].

## Conclusions

Workup of a palpable swelling in an EDS patient should include vascular pathology as well as soft tissue malignancies. Given vessel fragility, endovascular versus surgical approaches to correct vascular abnormalities in patients with EDS should be weighed against the risk of causing additional complications.
